# Effects of Resistance Exercise and Nutritional Supplementation on Dynamic Cerebral Autoregulation in Head-Down Bed Rest

**DOI:** 10.3389/fphys.2019.01114

**Published:** 2019-08-27

**Authors:** Marc Kermorgant, Nathalie Nasr, Marc-Antoine Custaud, Nastassia Navasiolava, Philippe Arbeille, Patrick Guinet, Marc Labrunée, Florent Besnier, Dina N. Arvanitis, Marek Czosnyka, Jean-Michel Senard, Anne Pavy-Le Traon

**Affiliations:** ^1^INSERM UMR 1048, Institute of Cardiovascular and Metabolic Diseases (I2MC), Toulouse, France; ^2^Department of Neurology, Institute for Neurosciences, Toulouse University Hospital, Toulouse, France; ^3^MITOVASC Institute, UMR CNRS 6015, UMR INSERM 1083, Clinical Research Centre, University Hospital of Angers, Angers, France; ^4^Faculty of Medicine, University of Tours, Tours, France; ^5^Department of Anesthesiology, Thoracic and Cardiovascular Surgery, Rennes University Hospital, Rennes, France; ^6^Department of Rehabilitation, Toulouse University Hospital, Toulouse, France; ^7^Brain Physics Laboratory, Division of Neurosurgery, Department of Clinical Neurosciences, Cambridge University Hospitals, Cambridge, United Kingdom; ^8^Institute of Electronic Systems, Warsaw University of Technology, Warsaw, Poland; ^9^Department of Clinical Pharmacology, Toulouse University Hospital, Toulouse, France

**Keywords:** cerebral autoregulation, head-down bed rest, microgravity, resistance vibration exercise, nutritional supplementation

## Abstract

Head-down bed rest (HDBR) is commonly considered as ground-based analog to spaceflight and simulates the headward fluid shift and cardiovascular deconditioning associated with spaceflight. We investigated in healthy volunteers whether HDBR, with or without countermeasures, affect cerebral autoregulation (CA). Twelve men (at selection: 34 ± 7 years; 176 ± 7 cm; 70 ± 7 kg) underwent three interventions of a 21-day HDBR: a control condition without countermeasure (CON), a condition with resistance vibration exercise (RVE) comprising of squats, single leg heel, and bilateral heel raises and a condition using also RVE associated with nutritional supplementation (NeX). Cerebral blood flow velocity was assessed using transcranial Doppler ultrasonography. CA was evaluated by transfer function analysis and by the autoregulatory index (Mxa) in order to determine the relationship between mean cerebral blood flow velocity and mean arterial blood pressure. In RVE condition, coherence was increased after HDBR. In CON condition, Mxa index was significantly reduced after HDBR. In contrast, in RVE and NeX conditions, Mxa were increased after HBDR. Our results indicate that HDBR without countermeasures may improve dynamic CA, but this adaptation may be dampened with RVE. Furthermore, nutritional supplementation did not enhance or worsen the negative effects of RVE. These findings should be carefully considered and could not be applied in spaceflight. Indeed, the subjects spent their time in supine position during bed rest, unlike the astronauts who perform normal daily activities.

## Introduction

Cerebral autoregulation (CA) allows the maintenance of constant cerebral blood flow (CBF) despite changes in blood pressure by adapting the vascular tone and cerebral vessel diameter (Nelson et al., 2014). CA plays a protective role to prevent capillaries from damage (Johnson, 1986). Several mechanisms may be involved in CA. These include metabolic influences (PaCO_2_, PaO_2_) and autonomic innervations (Paulson et al., 1990; Blaber et al., 2013; Zhang and Hargens, 2018). Available data indicate controversial CA adaptation after exposure to microgravity. Indeed, CA could be either impaired (Zhang et al., 1997) or improved (Jeong et al., 2014) when subjects spent a few weeks of bed rest. We recently demonstrated in healthy subjects that dynamic CA was improved after few days of dry immersion (Kermorgant et al., 2017). The same findings were reported during spaceflights. An impaired CA was described in astronauts during long duration flights on the International Space Station (Zuj et al., 2012). In contrast, a preserved or even enhanced CA was observed after short duration (Iwasaki et al., 2007) or long duration (Herault et al., 2000; Arbeille et al., 2001; Kotovskaia and Fomina, 2010) spaceflight.

Preventive countermeasures (i.e., drugs, nutritional countermeasures, and muscular exercise) to maintain crew health and fitness and to avoid the deconditioning associated with prolonged weightlessness have been tested in microgravity, yet most are only partially effective (Hargens and Richardson, 2009). Moreover, most of these countermeasures are time-consuming and may not be adapted to the schedule of the astronaut (Belin de Chantemele et al., 2004). In manned space missions, specific exercises combined with dietary intake represent the main countermeasures to sustain or restore fitness (maximal aerobic capacity, musculoskeletal structure, and orthostatic function). However, no single exercise, dietary regimen, or combination of prescription drugs have proven entirely effective in maintaining or restoring cardiovascular and musculoskeletal functions to preflight levels after prolonged space flight (Convertino, 2002).

In astronauts, resistance exercise was an effective countermeasure to avoid skeletal changes during long-duration spaceflight (Shackelford et al., 2004). Little is known about the impact of these countermeasures on CA in microgravity. However, it was shown that heavy resistance exercise induced decreased blood flow velocities likely due to the intense Valsalva maneuver and elevations in intracranial pressure in elite athletes (Dickerman et al., 2000). Furthermore, a previous study showed that during rhythmic resistance exercise that fluctuations in mean arterial pressure appear to be too rapid to be countered by CA (Edwards et al., 2002). Moreover, athletes who underwent dynamic resistance exercise showed temporary impairment in CA during the early recovery phase (Koch et al., 2005).

On the other hand, a diet high in protein led to a low-grade metabolic acidosis which altered bone metabolism after bed rest (Zwart et al., 2005). To neutralize these acidogenic effects of whey protein, several authors combined whey protein with potassium bicarbonate supplement in bed rest studies (Blottner et al., 2014; Bosutti et al., 2016). This combination remained partially effective on skeletal muscle atrophy (Blottner et al., 2014), but attenuated disuse-induced reductions in muscle fiber oxidative capacity (Bosutti et al., 2016). However, metabolic acidosis and potassium supplementation produce vasodilatory effects in cerebral arteries and vascular smooth muscle (Brayden, 1990; Nelson and Quayle, 1995; Kinoshita and Katusic, 1997; Ko et al., 2008) and these associated events may lead to an impaired CA. Indeed, several authors suggest that cerebral vasodilation could attenuate CA (Aaslid et al., 1989; Paulson et al., 1990; Panerai et al., 1999; Ito et al., 2003; Dineen et al., 2010).

This study was carried out in 12 healthy men who underwent three conditions during a 21-day of 6° head-down bed rest (HDBR): (1) a control condition without countermeasure (CON), (2) a condition with resistance vibration exercise (RVE), and (3) a condition with RVE and nutritional supplementation (NeX). HDBR is a ground-based analog to spaceflight that is commonly used to simulate headward fluid shift and cardiovascular deconditioning associated with spaceflight (Hargens and Vico, 2016). Therefore, the objective of this study was to determine whether RVE and NeX could alter CA after HDBR. We hypothesized that RVE and NeX may impair the effects of HDBR on CA by reducing cerebral perfusion.

## Materials and Methods

### Subjects

Twelve healthy male volunteers participated in this study (at selection: 34 ± 7 years; 176 ± 7 cm; 70 ± 7 kg).

The inclusion criteria were as follows:

• Healthy male volunteer, age between 20 and 45 years, height between 158 and 190 cm, with no overweight nor excessive thinness with BMI [weight (kg)/height (m^2^)] between 20 and 26.
• No personal nor family past record of chronic or acute disease or psychological disturbances which could affect the physiological data and/or create a risk for the subject during the experiment.
• Fitness level assessment:
  if age < 35 years: 35 ml/min/kg < V_O__2_ max < 60 ml/min/kg.
  if age > 35 years: 30 ml/min/kg < V_O__2_ max < 60 ml/min/kg.
• Active and free from any orthopedic, musculoskeletal, and cardiovascular disorders.
• No history of regular smoking, no alcohol, no drug dependence, and no medical treatment.

The exclusion criteria were as follows:

• Past record of orthostatic intolerance.
• Cardiac rhythm disorders.
• Chronic back pains.
• History of hiatus hernia or gastro-esophageal reflux, thyroid dysfunction, renal stones, diabetes, and migraines.
• Past records of thrombophlebitis, family history of thrombosis, or positive response in thrombosis screening procedure.
• Abnormal result for lower limbs echo-Doppler.
• History of genetic muscle and bone diseases of any kind.
• Bone mineral density: *T*-score ≤ −1.5.
• Osteosynthesis material, presence of metallic implants.
• History of knee problems or joint surgery/broken leg.
• Poor tolerance to blood sampling.
• Having given blood (more than 8 ml/kg) in a period of 8 weeks or less before the start of the experiment.
• History of intolerance to lactose or food allergy (milk proteins.)
• Positive reaction to any of the following tests: HVA IgM (hepatitis A), HBs antigen (hepatitis B), anti-HVC antibodies (hepatitis C), and anti-HIV1 + 2 antibodies.

This study (registered number: 2012-A00337-36) was carried out with the recommendations of the Ethics Committee (CPP Sud-Ouest Outre-Mer I). The protocol was approved by the French Health Authorities. All subjects gave written informed consent in accordance with the Declaration of Helsinki. The study was performed by the Institute for Space Medicine and Physiology (MEDES-IMPS) in Toulouse, France and supported by the French Spatial Agency [Centre National d’Etudes Spatiales (CNES)].

### General Protocol

**FIGURE 1:**
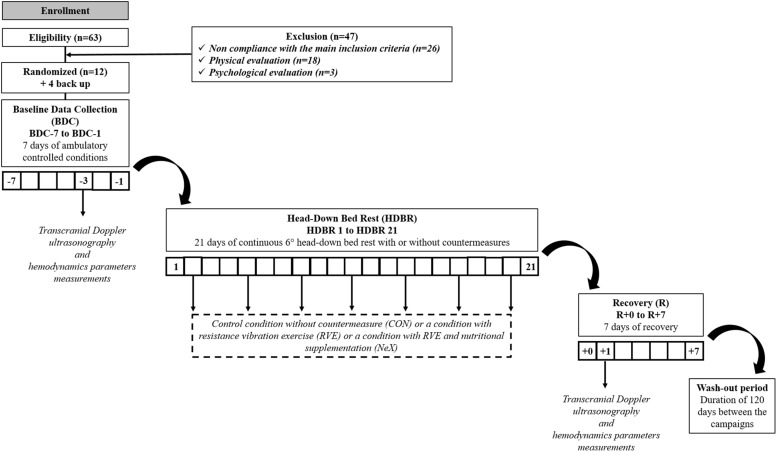
Flow chart of the study and timeline of data collection.

**FIGURE 2:**
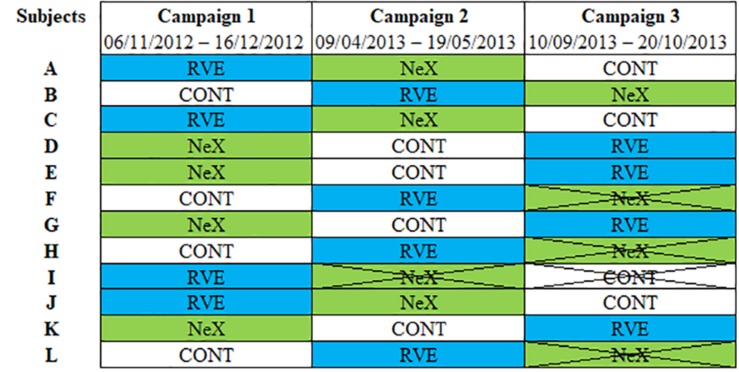
The different allocations for each subject with the exact dates of sessions. CON, control condition; NeX, resistance vibration exercise with nutritional supplementation condition; RVE, resistance vibration exercise condition.

Each volunteer underwent three hospital stays for duration of 35 days at MEDES-IMPS. Each hospitalization period included: (a) 7 days for ambulatory control period, (b) 21 days of HDBR period with or without countermeasures, (c) 7 days for recovery measurements after the HDBR period. A flow chart of one campaign is represented (Figure 1). The subjects were not allowed to get up or to sit up during HDBR. The order of the three interventions during HDBR was randomized: CON, RVE and NeX with the following sequences (CON/RVE/NeX or RVE/NeX/CON or RVE/CON/NeX). Four volunteers withdrew from the study, one during the second campaign and three others during the third campaign. The different allocations for each subject with the exact dates of sessions are presented in Figure 2. Another volunteer was excluded from the study due to low values of coherence (<0.5) in LF bandwidth for cross-spectral analysis. The three hospitalizations were performed over the same period for all participants. The wash-out period between the different hospitalization phases lasted 120 days.

Furthermore, 14 and 28 days after each sequence, subjects had to come back to MEDES-IMPS, for follow up visits. All exercise training was performed on an integrated training device supplied by Novotec Medical (Pforzheim, Germany). In this study, the vibration platform (Galileo^®^ Fitness, Novotec, Germany) was combined with a system designed to exercise in −6° lying position. Training was performed twice per week with 3–4 days intervals. The first training session was during the second day of HDBR.

### Resistance Vibration Protocol

During each session, the sequence was performed as follows:

• Warm up consisted to bilateral squats from 10° to 90° knee angle during 8 s (four down, four up) controlled by metronome with eight repetitions, load: 50% of the one repetition maximum (1-RM), amplitude: 8 mm, vibration frequency: 24 Hz.
• Bilateral squats from 10° to 90° knee angle during 8 s (four down, four up) controlled by metronome with 10 repetitions, load at study start: 75% of the 1-RM, progression: 5% load increase when more than 10 repetitions were possible, 5% load decrease when six or fewer repetitions were possible, amplitude: 8 mm, vibration frequency: 24 Hz.
• Single leg heel raises were carried out from maximal dorsiflexion to maximal plantar flexion as fast as possible until exhaustion, 1.3 times body weight (bw), progression: 5% load increase when more than 50 s were possible, 5% load decrease when 30 s or less were possible, amplitude: 8 mm, vibration frequency: 26 Hz.
• Bilateral heel raises were performed from maximal dorsiflexion to maximal plantar flexion as fast as possible until exhaustion, 1.8 times bw, progression: 5% load increase when more than 55 s were possible, 5% load decrease when 40 s or less were possible; amplitude: 8 mm, vibration frequency: 26 Hz.

### Nutritional Supplementation

An isocaloric supplementation of whey protein (0.6 g/kg bw/day) was given to the volunteers of the nutritional and exercise intervention sequence. The total protein intake was 1.8 g/kg bw/day. The schedule of the protein supplementation was the following: (1) on days without exercise, supplementation was applied in equal amounts with main meal and (2) on days with exercise, supplementation was divided and half of the daily amount in a timeframe of 30 min after exercise and the other half equally distributed with main meals. The product was Diaprotein^®^, a powder supplied by Nephrologische Präparate Dr. Volker Steudle (Linden, Germany). The composition was as follows: Diaprotein^®^ 100 g Powder, calorie intake 1573 kJ (370 kcal), proteins 90 g, fat 0.2 g, lactose 2.5 g, sodium < 300 mg, potassium < 650 mg, calcium < 400 mg, phosphorus < 250 mg, and relation phosphorus/protein < 3 mg/g. Since whey protein added a certain acid load to the diet, supplementation of 90 mmol potassium bicarbonate per day, applied in six portions (with main meals) was given to compensate for that. Potassium bicarbonate were provided by Krüger GmbH & Co. KG (Bergisch Gladbach, Germany). The amino acid profile of supplement is represented in Table 1.

**TABLE 1:** Detailed profile of the supplement.

	**Composition in%**
**Protein**	
β-lactoglobulin	45
α-lactalbumen	15
Bovine serum albumin	1.5
Immunoglobulin G	2
Lactoferrin	<1
Glycomacropeptide	26
Fat	1.20
Lactose	2.50
Ash	2.20
Sodium	0.25
Potassium	0.65
Calcium	0.40
Phosphorus	<0.25

### Transcranial Doppler Ultrasonography and Hemodynamics Parameters

**FIGURE 3:**
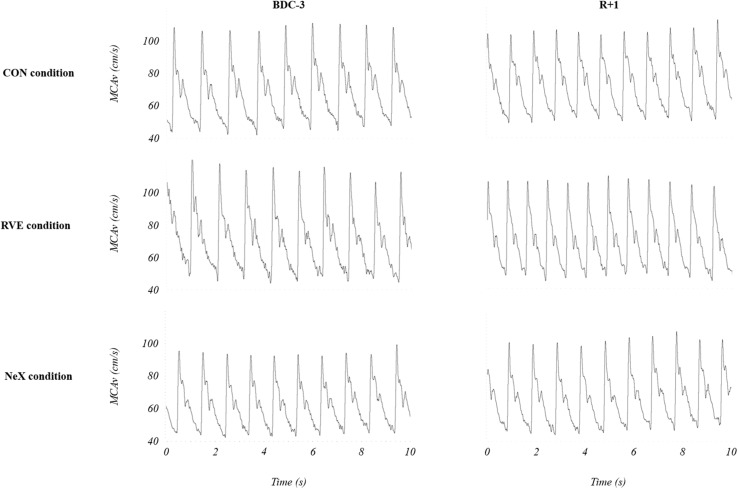
Representative transcranial Doppler data for each condition before (BDC-3) and after (*R* + 1) head-down bed rest. CON, control condition; NeX, resistance vibration exercise with nutritional supplementation condition; RVE, resistance vibration exercise condition.

Transcranial Doppler ultrasonography and hemodynamics parameters were performed at rest in supine position. The assessment of CA was conducted during the morning, 3 days before HDBR (BDC-3) and the first day of recovery (*R* + 1). A 2-MHz Doppler probe (EZ-DOP, DWL, Germany) maintained by a headset was fixed against the temporal window and insonated the signal from the middle cerebral artery to assess CBFV changes. The right middle cerebral artery was insonated unilaterally at a depth of 50–55 mm. The arterial blood pressure (ABP) was continuously monitored by photoplethysmography (Nexfin–B Meye, Netherlands). The signals CBFV and ABP were synchronized, acquired with Biopac MP 150 and visualized on the screen of a PC. The heart rate (HR) was measured continuously by an electrocardiogram (ADInstruments, Castle Hill, NSW, Australia). Representative data are provided for each condition (CON, RVE, and NeX) at BDC-3 and *R* + 1 in Figure 3.

### Assessment of Cerebral Autoregulation by Cross-Spectral Analysis

Beat-by-beat mean ABP and mean CBFV were linearly interpolated and resampled at 4 Hz for spectral analysis. Using fast Fourier transform with 50% superposition of segments (Welch algorithm), the mean ABP and mean CBFV time series were transformed from the time domain into the frequency domain. A length of 100 s was chosen for data segments and these segments were passed through a Hanning window. The transfer function analysis was used to study the relationship between changes in mean ABP and mean CBFV. To determine to what extent ABP influences on CBFV, a cross-spectral analysis method was applied to obtain estimates of the coherence, gain and phase. The coherence function measured the linearity of the relationship between input and output at a given frequency. A value close to 1 indicated, a strong linear relationship between the two signals with high signal-to-noise ratio, whereas the coherence approximating with values near zero may suggest a non-linear relationship, extraneous noise or other influencing variables (Marmarelis, 1988; Giller, 1990). The threshold of coherence over 0.5 was used to calculate the gain and phase shift (Diehl et al., 1995; Zhang et al., 1998). The gain of the transfer function reflected the efficiency of the CA and corresponds to the relative amplitude between the changes in ABP and CBFV signals. A low gain value represents an efficient CA (van Beek et al., 2008). The phase was considered as temporal relation between these signals (Zhang et al., 1998). The recordings with phase shift wrap-around were corrected by adding 2π. To assess dynamic CA, the mean values of the transfer function were calculated in very low frequency (VLF: 0.02–0.07 Hz) and low frequency (LF: 0.07–0.20 Hz) bands, as a measure of the transmission of blood pressure fluctuations on CBFV (Zhang et al., 1998, 2009). The mechanism of CA has the characteristics of a high-pass filter that dampens slow fluctuations of blood pressure (Zhang et al., 1998). The transfer function coherence (Giller, 1990; Donnelly et al., 2016), gain (Hamner et al., 2010; Donnelly et al., 2016), and phase (Immink et al., 2004; Donnelly et al., 2016) can provide valuable information on dynamic CA. Indeed, reduced coherence and gain associated with an elevated phase shift reflect an enhanced CA and only one of these modifications is sufficient to determine a better CA. These signals were processed with the NOTOCORD hem software (Notocord Systems, France).

### Assessment of Cerebral Autoregulation by the Autoregulatory Index

Cerebral autoregulation was assessed by autoregulatory index (Mxa) method established from the spontaneous slow variations of mean ABP and mean CBFV. ABP and CBFV signals were pre-processed with specific signal filtering to remove the presence of potential artifacts according to the recent recommendations (Claassen et al., 2016). Mxa indices were obtained from 30 consecutive 10-s periods between the mean ABP and mean CBFV previously calculated. Finally, the resulting 30 Mxa correlation coefficients were averaged in order to obtain the autoregulatory index Mxa value. When Mxa value approaches 1, ABP fluctuations affect changes in CBFV, indicating a defective CA. On the other hand, Mxa value nearly 0 shows that ABP variations are not associated with CBFV variations, suggesting a preserved CA (Czosnyka et al., 1996). A cut-off value of Mxa > 0.30 is considered to reflect an altered CA (Sorrentino et al., 2011).

### Statistics

Data were expressed as mean ± SD. We first checked whether data passed Shapiro–Wilk normality test. Mixed model for repeated measures with a Geisser–Greenhouse correction was used with Sidak’s multiple comparisons test. Subjects were entered as random factors and condition (CON, RVE, and NeX) and time (BDC-3 and *R* + 1) were included as fixed factors. All statistical analyses were performed with GraphPrism 8. Differences were considered as statistically significant when *P* < 0.05.

## Results

### Hemodynamic Parameters

There were no condition effects for systolic blood pressure (SBP) (*P* = 0.60), diastolic blood pressure (DBP) (*P* = 0.54), heart rate (HR) (*P* = 0.46), and middle cerebral arterial blood flow velocity (MCAv) (*P* = 0.51). There were no time effects for SBP (*P* = 0.83), DBP (*P* = 0.47), and MCAv (*P* = 0.43), whereas significant time effects were observed for HR (*P* = 0.02). There were no significant condition x time interactions for SBP (*P* = 0.18), DBP (*P* = 0.14), HR (*P* = 0.43), and MCAv (*P* = 0.43).

In CON condition, we noted no significant modifications either in SBP (*P* = 0.93) or DBP (*P* = 0.85) or MCAv (*P* = 0.25), whereas HR increased at *R* + 1 compared to BDC-3 (*P* < 0.01). In RVE condition, HR (*P* = 0.68) and MCAv (*P* = 0.89) did not change significantly at *R* + 1 compared to BDC-3, but we observed an upward trend in SBP (*P* = 0.17) and DBP (*P* = 0.13). In NeX condition, SBP (*P* = 0.91), DBP (*P* = 0.97), HR (*P* = 0.12), and MCAv (*P* = 0.96) did not change significantly at *R* + 1 compared to BDC-3 (Table 2).

**TABLE 2:** General hemodynamic parameters.

	**CON (*n* = 10)**		**RVE (*n* = 10)**		**NeX (*n* = 7)**	
	**BDC-3**	***R* + 1**	**BDC-3**	***R* + 1**	**BDC-3**	***R* + 1**
SBP (mmHg)	120 ± 7	118 ± 12	119 ± 10	125 ± 10	123 ± 10	120 ± 12
	(115–125)	(109–127)	(111–126)	(118–132)	(114–132)	(109–131)
DBP (mmHg)	68 ± 4	70 ± 7	68 ± 3	72 ± 5	72 ± 8	71 ± 4
	(65–71)	(65–75)	(66–70)	(69–76)	(65–79)	(67–74)
HR (bpm)	57 ± 10	64 ± 11^∗∗^	60 ± 10	63 ± 5	57 ± 11	63 ± 10
	(50–64)	(57–72)	(53–67)	(60–67)	(46–67)	(54–72)
MCAv (cm/s)	61 ± 5	65 ± 9	60 ± 7	63 ± 10	61 ± 6	59 ± 7
	(57–65)	(59–72)	(55–66)	(55–70)	(55–67)	(53–65)

*CON, control condition; DBP, diastolic arterial blood pressure; HR, heart rate; MCAv, middle cerebral artery velocity; NeX, resistance vibration exercise with nutritional supplementation condition; RVE, resistance vibration exercise condition; SBP, systolic arterial blood pressure. ^∗∗^*P* < 0.01 vs. BDC-3 in CON condition. Values in parentheses represent 95% CI.*

### Analysis of Cerebral Autoregulation

In VLF bandwidth, the gain and phase could not be taken into account because the coherence value was below 0.5.

There were no condition effects for coherence (*P* = 0.57), gain (*P* = 0.36), phase (*P* = 0.37), and Mxa (*P* = 0.06). There were significant time effects for Mxa (*P* < 0.01) but not for coherence (*P* = 0.20), gain (*P* = 0.41) and phase (*P* = 0.77). There were significant condition × time interactions for Mxa (*P* < 0.001) but not for coherence (*P* = 0.17), gain (*P* = 0.22), and phase (*P* = 0.31).

Coherence was increased in RVE condition (*P* = 0.02) but remained unchanged in CON (*P* = 0.96) and NeX (*P* = 0.86) conditions at *R* + 1 compared to BDC-3. Gain was not significantly modified in CON (*P* = 0.90), RVE (*P* = 0.74), and NeX (*P* = 0.56) conditions at *R* + 1 compared to BDC-3. Phase was not significantly changed in CON (*P* = 0.94), RVE (*P* = 0.50), and NeX (*P* = 0.99) conditions at *R* + 1 compared to BDC-3. In CON condition, Mxa index was significantly reduced at *R* + 1 compared to BDC-3 (*P* < 0.001). On the contrary in RVE and in NeX conditions, Mxa was significantly increased at *R* + 1 compared to BDC-3 (respectively *P* = 0.03 and *P* < 0.01) (Table 3).

**TABLE 3:** Analysis of cerebral autoregulation.

	**CON (*n* = 10)**		**RVE (*n* = 10)**		**NeX (*n* = 7)**	
	**BDC-3**	***R* + 1**	**BDC-3**	***R* + 1**	**BDC-3**	***R* + 1**
Coherence	0.67 ± 0.13	0.65 ± 0.15	0.64 ± 0.11	0.75 ± 0.10^†^	0.67 ± 0.16	0.71 ± 0.12
	(0.58–0.76)	(0.54–0.76)	(0.55–0.71)	(0.68–0.82)	(0.53–0.82)	(0.60–0.81)
Gain (cm/s/mmHg)	0.75 ± 0.15	0.72 ± 0.17	0.71 ± 0.15	0.77 ± 0.14	0.73 ± 0.15	0.85 ± 0.16
	(0.64–0.86)	(0.60–0.83)	(0.60–0.82)	(0.67–0.87)	(0.59–0.87)	(0.70–1.00)
Phase (rad)	0.93 ± 0.33	0.99 ± 0.29	1.06 ± 0.20	0.93 ± 0.33	0.95 ± 0.25	0.97 ± 0.24
	(0.70–1.15)	(0.78–1.20)	(0.91–1.20)	(0.70–1.17)	(0.72–1.18)	(0.75–1.19)
Mxa	0.26 ± 0.06	0.15 ± 0.06^∗∗∗^	0.22 ± 0.16	0.38 ± 0.10^†^	0.21 ± 0.15	0.41 ± 0.13^‡‡^
	(0.22–0.30)	(0.11–0.20)	(0.11–0.34)	(0.31–0.46)	(0.08–0.35)	(0.29–0.53)

*CON, control condition; NeX, resistance vibration exercise with nutritional supplementation condition; RVE, resistance vibration exercise condition. ^∗∗∗^*P* < 0.001 vs. BDC-3 in CON condition, ^†^*P* < 0.05 vs. BDC-3 in RVE condition, ^‡‡^*P* < 0.01 vs. BDC-3 in NeX condition. Values in parentheses represent 95% CI.*

## Discussion

Our findings indicate that dynamic CA was improved after 21 days of HDBR in healthy subjects. These results indicate that there are no abnormalities in dynamic CA after HDBR; further suggesting that cerebral mechanisms preserved CBF. We also showed that dynamic CA was impaired by RVE. Whey protein plus potassium bicarbonate supplement did not improve or exacerbate the negative effects of RVE.

The CON condition, without any countermeasure, did not induce significant changes in SBP and DBP. This steady state of blood pressure was mainly due to the increase in HR provoked by the baroreflex activation due to the fluid shift (Custaud et al., 2002). We found in CON condition, a reduction in Mxa values indicating an enhancement in CA. Usually, the assessment of CA by cross-spectral analysis is evaluated in VLF (0.02–0.07 Hz) and LF bandwidths (0.07–0.20 Hz) as previously described (Claassen et al., 2016). However, in our study, the VLF coherence value was below 0.5, so the gain and phase values were not sufficiently robust. Studies about microgravity exposure reported an impaired, unmodified or enhanced CA. Indeed, six healthy male volunteers had a gradual decrease in CBFV during a 21-day HDBR depicting an altered CA (Sun et al., 2005). Nevertheless, a previous study performed in women during a 7-day HDBR depicted no major modification in parameters of transfer function analysis and consequently, no significant alteration in dynamic CA (Pavy-Le Traon et al., 2002). Other findings showed the same trends during an acute head-down tilt test, where transfer function magnitude and phase angle between mean ABP and mean CBFV were not modified (Cooke et al., 2003). After a 3-day dry immersion study, another ground-based analog to microgravity, we found a reduction in Mxa values accompanied by an elevation in LF transfer function phase indicating an enhancement in dynamic CA (Kermorgant et al., 2017). Furthermore, astronauts exhibited an improved dynamic CA compared with preflight values; LF gain was significantly reduced after 1- and 2-week space mission (Iwasaki et al., 2007). One assumption is that the reduction in plasma volume could partly explain the CA improvement. Despite the lack of plasma volume data in our study, it was commonly described that plasma volume was reduced during shorter duration study (Vernikos et al., 1993; Convertino, 1996; Custaud et al., 2002; Jeong et al., 2014). Indeed, some studies performed in healthy subjects showed that an acute hypovolemia decreased transfer function gain between spontaneous changes of mean ABP and mean CBFV, thus describing an improved CA. Conversely, when plasma volume was restored by volume loading, this transfer function gain was increased indicating an impaired CA (Ogawa et al., 2007, 2009; Jeong et al., 2014). At present, the mechanisms by which changes in plasma volume affects dynamic CA remain unclear. Arterial partial pressure of CO_2_ (PaCO_2_) may also affect CBF. Indeed, it has been demonstrated that PaCO_2_ is a strong regulator of CBF (Claassen et al., 2007; Peebles et al., 2007; Sato et al., 2012; Brothers et al., 2014; Skytioti et al., 2018). It is well known that hypocapnia induces cerebral vasoconstriction so may enhance CA, while hypercapnia induces cerebral vasodilation so could attenuate CA (Aaslid et al., 1989; Paulson et al., 1990; Panerai et al., 1999; Ito et al., 2003; Dineen et al., 2010). Unfortunately, due to technical problems, PaCO_2_ could not be assessed in our study. However, previous data demonstrated that CBF was maintained through CA in the face of superimposed steady-state orthostatic stress and dynamic changes in PaCO_2_ (Day et al., 2013). Other findings suggested that during steady-state orthostatic stress, the ability of subjects to mount a normal ventilatory response to increased CO_2_ was unaffected, despite any potential changes in pulmonary mechanics associated with positional challenges (Skow et al., 2014). Another hypothesis for this CA improvement is that HDBR could raise the responsiveness of cerebral vascular smooth muscle to changes of transmural pressure (Iwasaki et al., 2007).

In RVE condition, transfer function coherence in LF range and Mxa values were higher after HDBR. Indeed, the specific characteristic of cerebrovascular resistance suggests that coherence values should be low in normal conditions and high when CA is disturbed (Panerai, 1998). Higher Mxa values are considered as an impairment in CA (Donnelly et al., 2016). Thus, the present results indicate a potential impairment in CA after HDBR associated with RVE countermeasure. We also observed an upward trend in SBP (from 119 to 125 mmHg) and DBP (from 68 to 72 mmHg). It is well known that mean cerebral perfusion pressure is dependent upon mean ABP (Schmidt et al., 2001; Czosnyka and Pickard, 2004). However, the normal range of dynamic CA is defined approximately for mean ABP values between 60 and 160 mmHg (Armstead, 2016; Kinoshita, 2016). Therefore, in this case, dynamic CA should not be impaired for this slight elevation in ABP in RVE condition. During resistance exercise, however, evidence indicates that CBF decreases likely due to the intense Valsalva and elevations in intracranial pressure (Dickerman et al., 2000; Tzeng and Ainslie, 2014). A previous study reported that fluctuations in CBF during rhythmic resistance exercise indicates that fluctuations in ABP with each muscle contraction can be too rapid to be countered by CA (Edwards et al., 2002). A study performed in 16 female and 16 male athletes proved that dynamic resistance exercise could temporarily impair dynamic CA during the early recovery phase (Koch et al., 2005). In our study, plasma volume was unlikely to be changed during RVE condition. Indeed, a previous study reported that during a 90-day HDBR, resistance exercise does not prevent the decrease in plasma volume (Belin de Chantemele et al., 2004). Therefore, variations of plasma volume may not be sufficient to explain the alterations found in CA. It is well known that intracranial pressure affects dynamic CA (Petersen and Ogoh, 2019). However, in our study, intracranial pressure is unlikely to be changed after RVE. Indeed, Lefferts et al. (2015) showed that there were no changes in intracranial pressure (measured by optic nerve sheath diameter, a surrogate marker of intracranial pressure) following acute high-intensity resistance exercise. One explanation to this impaired CA is that RVE would lead to an alteration in cerebral transmural pressure (Lefferts et al., 2015). Although the threshold usually used to consider impaired CA is Mxa > 0.30 (Sorrentino et al., 2011), the absolute value of these thresholds remains uncertain, and Mxa should be considered a continuous index that reflects a wide spectrum of severity of CA impairment (Crippa et al., 2018). Given that the Mxa threshold is not well established, CA could be considered impaired by RVE but not in terms of pathologic.

In NeX condition, Mxa values were higher after HDBR. Taken together, whey protein plus potassium bicarbonate supplement did not improve or worsen the negative effects of RVE on CA in our study. Previous studies have aimed to determine the impact of whey protein plus potassium bicarbonate supplement on disused muscle after bed rest; however, the efficiency of this countermeasure remains partial with limited impact on skeletal muscle atrophy (Blottner et al., 2014), even if it appears to attenuate disuse-induced reductions in muscle fiber oxidative capacity (Bosutti et al., 2016). Moreover, it has been shown that a diet high in protein may lead to a low-grade metabolic acidosis (Zwart et al., 2005). Therefore, to counteract the possible occurring low-grade metabolic acidosis, an additional supplementation with potassium bicarbonate was applied in this study, as described in previous studies (Blottner et al., 2014; Bosutti et al., 2016). Kinoshita and Katusic (1997) showed that acidosis may induce a pH−dependent vasodilation of isolated canine basilar arteries and potassium supplementation may elicit vasodilation of the vascular smooth muscle (Brayden, 1990; Nelson and Quayle, 1995; Ko et al., 2008) and these events may lead to an impaired CA. Indeed, as previously mentioned, several authors showed that cerebral vasodilation could attenuate CA (Aaslid et al., 1989; Paulson et al., 1990; Panerai et al., 1999; Ito et al., 2003; Dineen et al., 2010). The impact of metabolic acidosis on CA is not well known; however, it would seem that CA would be preserved in the newborn dogs in this situation (Hermansen et al., 1984). The involvement of potassium in the cerebral vascular smooth muscle relaxation is unclear and it is considered that potassium alone could not be responsible for the relaxation in vascular smooth muscle (Zhang and Cook, 1994). Furthermore, the response to potassium supplementation is slow to appear, taking longer than 4 weeks (Matlou et al., 1986; Haddy et al., 2006; Okazaki et al., 2009). This may explain why whey protein plus potassium bicarbonate supplementation did not enhance or worsen the negative effects of RVE on CA in our 21-day HDBR study.

### Study Limitations

The study was performed on a limited number of subjects, which could have dampened the statistical significance of our results; this was though limited by the fact that the subjects were their own controls. We considered that the diameter of the MCA at a depth of 50–55 mm was constant to properly assess CBFV and this potential issue is inherent to CA studies using transcranial Doppler; however, this technique is still widely used to assess CA (Nasr et al., 2014). The supine position is not the optimal position to examine dynamic CA and may influence the transfer function analysis, since coherence will remain low. Indeed, significant differences could be noticed whether experiments are performed in supine or seated position (Saul et al., 1989; Smirl et al., 2015). Moreover, CO_2_, which is a potent cerebral vasodilator, influences CA, was not monitored during HDBR. Nevertheless, Mxa was calculated over several minutes that may minimize the impact of episodic CO_2_ on CBFV. Plasma volume should have been carried out, since plasma volume variations may impact on CA (Jeong et al., 2014). Taking into account the influence of gender on CA (Deegan et al., 2009), as well as regional differences (anterior vs. posterior cerebral circulation) (Roth et al., 2017) in several CBF determinants, these results cannot be generalized to women or the posterior cerebral circulation. Finally, the conclusions made in our study should be carefully considered. Note that each specific metric used to assess CA is independent and these metrics do not correlate with each other (Tzeng et al., 2012).

In summary, these results suggest that dynamic CA was improved after 21 days of HDBR. However, this improvement could be dampened with RVE. Moreover, nutritional supplementation did not enhance or worsen the negative effects of RVE on CA. Finally, the development of optimized exercise and nutritional intake need to be further studied in order to assess and mitigate the potential negative effects of these countermeasures on CA. Indeed, an impaired CA has been proposed as a contributing factor to post-spaceflight orthostatic intolerance. However, these conclusions should be interpreted cautiously, because these findings could not be applied in spaceflight. Indeed, the subjects spent their time in supine position during bed rest, unlike the astronauts who perform normal daily activities.

## Data Availability

All datasets generated for this study are included in the manuscript and/or the supplementary files.

## Ethics Statement

This study was carried out in accordance with the recommendations of the 18th World Medical Assembly (Helsinki, 1964) and approved by the Ethics Committee (CPP Sud-Ouest Outre-Mer I) with written informed consent from all subjects. All subjects gave written informed consent in accordance with the Declaration of Helsinki. The protocol was approved by the French Health Authorities.

## Author Contributions

M-AC, NatN, PA, PG, and AP-LT designed the research. M-AC, NatN, NasN, PA, PG, and AP-LT performed the research. MK, NatN, M-AC, NasN, PA, J-MS, and AP-LT analyzed the data. MK, M-AC, NatN, ML, FB, PA, PG, DA, MC, J-MS, and AP-LT wrote the manuscript.

## Conflict of Interest Statement

The authors declare that the research was conducted in the absence of any commercial or financial relationships that could be construed as a potential conflict of interest.
